# Adapted Low-FODMAP Diet in IBS Patients with and without Fibromyalgia: Long-Term Adherence and Outcomes

**DOI:** 10.3390/nu16193419

**Published:** 2024-10-09

**Authors:** Christian Lambiase, Alessandra Rossi, Riccardo Morganti, Lorenzo Cancelli, Antonio Grosso, Riccardo Tedeschi, Francesco Rettura, Marta Mosca, Nicola de Bortoli, Massimo Bellini

**Affiliations:** 1 Gastrointestinal Unit, Department of Translational Research and New Technologies in Medicine and Surgery, University of Pisa, 56124 Pisa, Italy; 2 National Institute for Health and Care Research Nottingham Biomedical Research Centre, Nottingham University Hospitals NHS Trust and the University of Nottingham, Nottingham NG7 2UH, UK; 3 Nottingham Digestive Diseases Centre, Translational Medical Sciences, School of Medicine, University of Nottingham, Nottingham NG7 2UH, UK; 4 Rheumatology Division, Department of Clinical and Experimental Medicine, University of Pisa, 56126 Pisa, Italy; 5 Clinical Trial Statistical Support Unit, Azienda Ospedaliero Universitaria Pisana, 56126 Pisa, Italy; 6 Gastroenterology Unit, Annunziata Hospital, 87100 Cosenza, Italy

**Keywords:** FODMAP, low-FODMAP diet, irritable bowel syndrome, fibromyalgia

## Abstract

Background/Objectives: A low-FODMAPs Diet (LFD) is considered a “second line” dietary strategy for irritable bowel syndrome (IBS) but, after a period of strict restriction of all FODMAP foods, it has to be adapted and tailored to each patient (AdLFD). Fibromyalgia often coexists with IBS in up to 65% of cases. Our aims were to evaluate if comorbid fibromyalgia influenced the long-term clinical outcomes and adherence to an AdLFD in IBS patients. Methods: IBS patients with or without fibromyalgia who had started an AdLFD were enrolled. Patients had been evaluated before starting the LFD (T0). After a mean follow-up of 62.5 ± 22.7 months (T1), they were re-evaluated using questionnaires on disease severity, bowel habits, psychological status, and adherence to AdLFD. Results: In total, 51 IBS patients entered the study. Nineteen of them had comorbid fibromyalgia. Thirty patients reported a reduction in symptom severity at T1 in comparison with T0. Despite some slight differences in single IBS Symptom Severity Score items, comorbid fibromyalgia did not influence the IBS-SSS total score at T1. Patients with comorbid fibromyalgia showed a higher Hospital Anxiety and Depression Scale (HADS) score at baseline. A total of 44 patients showed good long-term adherence to the AdLFD. All patients improved their HADS score and had long-term adherence to the AdLFD. Conclusions: Comorbid fibromyalgia showed only a slight influence on long-term outcomes of an AdLFD on IBS symptoms, without affecting the relief of global symptoms. No influence on long-term adherence to AdLFD was detected. Hence, this approach can be taken into account in fibromyalgia patients for a nonpharmacological management of IBS symptoms. However, multicentric studies on larger samples would be welcome in the future.

## 1. Introduction

Irritable bowel syndrome (IBS) is one of the most common disorders of gut–brain interaction, with a prevalence of about 4% in the general population [1]. It is characterized by recurrent episodes of abdominal pain associated with changes in bowel habits, stool consistency, and frequency [2]. Currently, international guidelines suggest making a positive diagnosis of IBS based mainly on symptoms and performing very few tests (in the absence of alarm features) [3,4]. IBS often overlaps with other disorders of gut–brain interaction [5], anxiety and depression [6], chronic fatigue [7], and other chronic pain conditions, such as fibromyalgia [8,9,10,11]. Currently, the etiology of both fibromyalgia and IBS is not fully understood, although there is evidence supporting common pathogenetic mechanisms [12,13,14,15,16].

The diagnosis of fibromyalgia, as well as IBS, is based on symptoms and is made in the presence of widespread pain and tender points, fatigue, sleep disturbances, and a general increase in somatic complaints [17]. The prevalence of fibromyalgia in IBS ranges from 20 to 65% and, among fibromyalgia patients, up to 81% may have IBS symptoms [18]. IBS and fibromyalgia are both more prevalent in women and their pathogenesis involves alterations in the functioning of the sympathetic nervous system, with resulting central sensitization [8,10]. Furthermore, comorbid fibromyalgia is associated with more severe IBS symptoms [12]. Both fibromyalgia and IBS are also frequently associated with psychological comorbidities: prevalence of depressive and anxiety disorders were reported in 20–80% and 13–63.8% of fibromyalgia patients, respectively [19], and up to 39% of IBS patients report a depressive and/or anxious pathological state [20].

Diet plays a crucial role in the pathophysiology of IBS, with clear correlations between the intake of certain foods (i.e., FODMAPs (“Fermentable Oligosaccharides, Disaccharides, Monosaccharides and Polyols”)) and the onset of gastrointestinal symptoms [21,22,23]. A low-FODMAP diet (LFD) is currently considered a dietary strategy to treat IBS [3,4,24] and there is emerging evidence that it may have a role also in the management of other disorders of gut–brain interaction, such as fecal incontinence [25] or functional dyspepsia [26] and in other chronic pain conditions, such as fibromyalgia [27,28,29].

The aims of our study were to evaluate, in a group of IBS patients with and without fibromyalgia (IBSF and IBSWF, respectively), whether it was possible to find any difference regarding the long-term adherence to an AdLFD and its clinical outcomes.

## 2. Materials and Methods

### 2.1. IBS Patients with or without Fibromyalgia

IBS patients were evaluated at the outpatient service of the Gastrointestinal Unit of the University of Pisa. A positive IBS diagnosis was made according to the Rome IV diagnostic criteria [2]. Based on clinical history and physical examination, if the gastroenterologist suspected a possible overlapping fibromyalgia, patients were referred to the Division of Rheumatology of the University of Pisa for confirmation or exclusion of fibromyalgia. Fibromyalgia diagnosis was made on the basis of the 2016 revised fibromyalgia diagnostic criteria by a rheumatologist [17].

A total of 105 IBS patients (89 females and 16 males), both with comorbid fibromyalgia (IBSF) and without comorbid fibromyalgia (IBSWF), were referred for the LFD prescription to a skilled nutritionist for the management of IBS symptoms. A total of 41 patients discontinued the diet because it was not effective and 64 patients proceeded to the adapted phase of the LFD. In total, 51 patients out of 64 were reassessed to evaluate the long-term outcomes and adherence (Table 1). All patients were over 18 years old and were consecutively enrolled starting from September 2016.

### 2.2. Study Design

Subjects with established or presumed organic or psychiatric conditions potentially interfering with the study were excluded from the study, along with those who had regularly used laxative or other medications with effects on abdominal pain, abdominal discomfort, bloating, or abdominal distension in the four weeks before enrollment. Anthropometrical measurements consisting of height, weight, and body mass index (BMI) were collected according to the international criteria [30].

Before starting the LFD, all patients received a comprehensive initial evaluation (T0) as part of their clinical management. At T0, a careful clinical and pharmacological history, focused on personal and behavioral habits, was collected through a structured interview questionnaire. A dietary history was collected by a skilled nutritionist through the analysis of a previously completed food diary and by means of targeted questions regarding feelings of hunger and satiety, food preferences or aversions, and any intolerances and allergies. At T0, patients were evaluated by the IBS Symptom Severity Score (IBS-SSS) [31], the Bristol Stool Form Chart (BSFC) [32], and a “homemade” bowel habits questionnaire, already used in previous studies [33]. The Hospital Anxiety and Depression Scale (HADS) [34] was also administered to all patients at T0 to record their levels of anxiety or depression. After 8 weeks of LFD, responder patients proceeded to the adapted LFD (AdLFD). Patients who had begun an AdLFD at least 6 months before were reassessed in January 2024 through a telephone consultation (T1). The following questionnaires were administered again to the patients: the IBS-SSS, the BSFC, the “bowel habits questionnaire”, and the HADS. Furthermore, at T1, the FODMAP Adherence Report Scale (FARS) [35], a “degree of relief from symptoms” Likert scale, and a “degree of treatment satisfaction” scale were also administrated to the patients [33,36,37].

This study was conducted according to the ethical principles of the Declaration of Helsinki and to good clinical practice (GCP). Final approval was granted by the Ethics Committee of North-West Tuscany (study number 1136/2016).

### 2.3. Questionnaires

The IBS Symptom Severity Score (IBS-SSS) [31] is a score calculated to assess the intensity of IBS symptoms based on five items: severity of abdominal pain, number of days with abdominal pain, severity of abdominal bloating/distention, satisfaction with bowel habits, and IBS-related quality of life (QoL). Each item is scored on a visual analogue scale ranging from 0 to 100. A score of less than 75 points is considered normal/remission and the maximum score is 500. A score between 75 and 175 is considered mild IBS, a score between 176 and 300 is considered moderate IBS, and a score > 300 is considered severe IBS. Within this system, there is a definition of “response” as an improvement in total IBS-SSS of at least 50 points [31].

The Hospital Anxiety and Depression Scale (HADS) [34] is a validated screening tool used to assess anxiety (HAD-A) and depression (HAD-D) levels in patients. It has been previously used to evaluate anxiety and depression in IBS outpatient patients [33,38].

The “bowel habits questionnaire” [33] is a ‘‘homemade’’ questionnaire and was used to evaluate the frequency of the following symptoms associated with defecation: straining at defecation, incomplete evacuation, painful defecation, hard stools (BSFC 1–2), watery stools (BSFC 6–7), “fragmented” defecation, defecatory urgency, incontinence for gas and/or feces, abdominal pain, and abdominal bloating. It uses a scale to indicate frequency of symptoms associated with bowel movements: 0 = no symptom associated with bowel movements, 1 = symptoms occur during <25% of bowel movements, 2 = symptoms occur between 25 and 49% of bowel movements, 3 = symptoms occur between 50 and 75% of bowel movements, and 4 = symptoms occur during >75% of bowel movements.

The FARS [35] was used to evaluate the degree of the patient’s adherence to the AdLFD. It consists of 5 questions, each with five possible answers (always, often, sometimes, rarely, and never) to which a score ranging from 1 to 5, respectively, is assigned. FARS has a maximum score of 25 points. A total score of at least 20 points (≥80%) is considered as a sign of good adherence to the diet [35].

The “degree of relief” is a Likert scale that was used to estimate the symptom improvement perceived by the patient compared to the basal values. The answer is indicated on an 8-point visual analogue scale ranging from 0 to 7, where 7 means that the patient feels much worse than at the beginning of the treatment, 4 means nothing has changed, and 0 indicates that the patient feels completely relieved compared to the beginning of the study, with complete remission of symptoms [36,37].

The “degree of treatment satisfaction” scale was used to estimate patients’ long-term satisfaction with the AdLFD. The patient was asked to mark the answer on an 11-point visual analogue scale, where 0 indicates that the patient was totally dissatisfied with the diet and 10 indicates the patient was completely satisfied [36,37].

### 2.4. LFD and AdLFD

The LFD was carried out by an expert nutritionist, as described in our previous work [33] (Table S1). Briefly, at T0, a strict LFD, according to Shepard and Gibson indications, was prescribed to the patient [39]. The LFD was recommended for 8 weeks [39]. To increase adherence to the diet and ensure nutritional adequacy in terms of carbohydrates, protein, lipids, minerals, and vitamins, the diet’s composition was customized for each patient. In this period the nutritionist performed a telephone consultation every 2 weeks in order to resolve any issues related to the dietary management. Patients who did not respond to the strict LFD did not proceed to the LFD reintroduction phase.

FODMAPs were reintroduced one category at a time for four days, according to Shepard and Gibson [39], to detect the “trigger” foods that were responsible for the symptoms. The target was to identify the foods containing FODMAPs that the patient was able to consume without having an exacerbation of gastrointestinal symptoms. This enabled the nutritionist to suggest a long-term and less strict diet (i.e., AdLFD), customized for each patient based on different FODMAP intolerance. After a variable minimum period of 6 months from the beginning of the AdLFD, patients were invited to a new nutritional evaluation as part of standard clinical practice.

### 2.5. Statistical Analysis

Categorical data were described with absolute and relative (%) frequency and continuous data were summarized with mean and standard deviation. To analyze categorical and continuous data, the chi square test or Fisher’s exact test and *t*-test for independent samples or one-way ANOVA (when appropriate) were applied, respectively. Repeated measures of continuous variables were compared by *t*-test for paired data and ANOVA for repeated measures. Finally, to evaluate the factors influencing the response (no, yes), a multivariate binary logistic model using the stepwise method was used and OR with a 95% CI was also indicated. Significance was set at 0.05 and all analyses were carried out with SPSS v.29 technology.

## 3. Results

### 3.1. Patients

Overall, starting from September 2016, a total of 105 IBS patients who had undergone an LFD for management of IBS symptoms were considered for the enrollment. Forty-one patients discontinued the diet because they did not have any beneficial effects during the first 8 weeks of the LFD (strict phase). Sixty-four patients (60.95%) proceeded to the adapted phase of the LFD. There were no differences regarding the frequency of comorbid fibromyalgia, IBS subtype, age, or gender between patients who did and did not respond to the strict LFD. In January 2024, patients were contacted again through a telephone interview to reassess symptoms, AdLFD outcomes, and adherence. A total of 51 patients out of 64 (79.69%) answered the telephone call and agreed to proceed with the telephone consultation. These patients were enrolled for data analysis. Thirteen patients were considered lost at follow-up, mainly because no answer was obtained after multiple attempts to telephone or because they refused to answer the telephone interview (time-related issues, privacy reasons, etc.). There were no differences regarding the frequency of comorbid fibromyalgia, IBS subtype, age, or gender between patients who did and did not undergo the telephone interview at T1.

Patients’ mean age was 53.1 ± 13.3 years and the mean BMI was 24.5 ± 4.2 kg/m^2^. A total of 32 patients out of 51 (62.7%) showed only IBS (IBSWF), whereas 19/51 patients (37.3%) had an overlapping IBS–fibromyalgia (IBSF). Based on predominant stool consistency, 23 patients showed IBS with predominant diarrhea (IBS-D), 8 patients IBS with predominant constipation (IBS-C), and 20 patients IBS with mixed bowel habits (IBS-M). IBSWF and IBSF patients showed no differences regarding clinical subtypes of IBS (Table 1). The demographic characteristics of enrolled patients are summarized in Table 1. No differences were observed in mean age, gender, or BMI comparing IBSF and IBSWF groups (Table 1).

The mean follow-up was 62.5 ± 22.7 months. No differences in the length of mean follow-up were observed between IBSF and IBSWF groups (*p* = 0.89). IBS-C patients showed a longer follow-up (78.8 ± 9.5 months) compared to IBS-D patients (58.0 ± 26.1 months) (*p* = 0.002).

The most frequently excluded FODMAP foods in the long term were those containing lactose (80.4%), fructose (58.8%), and fructans (62.7%), while only 17.6% and 23.5% of patients excluded foods containing galactans or polyols in the long term, respectively (Table S2). There were no differences in food exclusions between IBSF and IBSWF groups (Table S2).

### 3.2. Clinical Outcomes of Long-Term AdLFD

#### 3.2.1. IBS-SSS

Based on the IBS-SSS score at T0, 3 patients showed a mild IBS (5.9%), 20 patients showed a moderate IBS (39.2%), and 28 patients showed a severe IBS (54.9%) (Table 2). Thirty patients (58.8%) reported an improvement in total IBS-SSS score ≥ 50 points at T1 in comparison to T0 and, therefore, they can be considered “long-term responders” to AdLFD. Regarding the severity of IBS, at T1:

- Four patients (7.8%) were in remission (IBS-SSS total score < 75) (two of them had a severe IBS and two a moderate IBS at T0).
- Fifteen patients (29.4%) showed a mild IBS (five of them had a moderate IBS and nine of them a severe IBS at T0; one patient did not improve or worsened).
- Twenty patients (39.2%) showed a moderate IBS (ten of them had a severe IBS at T0; eight patients did not improve or worsened and two patients who had mild IBS at T0 showed a moderate IBS at T1).
- Twelve patients (23.5%) showed a severe IBS (seven patients did not improve and five patients showed a worsening of symptoms and changed from a moderate to severe IBS).

At T0 and T1, the IBSF and IBSWF groups showed a similar IBS-SSS total score and IBS-SSS items (Table 2). The mean IBS-SSS total score at T0 was 313.5 ± 95. Comorbid fibromyalgia had no impact on IBS severity response evaluated through total IBS-SSS score [31]. There was a significant improvement in the mean IBS-SSS total score at T1 (220 ± 112, *p* < 0.001) considering all patients and IBSF and IBSWF groups. Regarding IBS-SSS items, considering all IBS patients, at T1, there was an improvement in abdominal pain severity, days with abdominal pain, bowel habit dissatisfaction, and IBS interference with lifestyle (Table 2). There was also an improvement in abdominal distention severity between T0 and T1, although not statistically significant. IBSWF patients reported significant improvement in the abdominal distension severity, while IBSF patients did not. Similarly, IBSF patients showed significant improvement in the interference of IBS with lifestyle if compared to IBSWF patients. However, comparing the improvement rates between IBSF and IBSWF for each IBS-SSS item, there were no statistically significant differences (Table 2).

#### 3.2.2. Degree of Symptom Relief and Degree of Treatment Satisfaction

The degree of relief of symptoms, evaluated on a Likert scale ranging from 0 to 7, showed an improvement in symptoms at T1 (1.6 ± 1.7). Forty-one patients (80.4%) reported scores of between 0 and 3. There was no difference in symptom relief between IBSWF and IBSF groups and in different IBS subtypes (*p* = 0.285 and *p* = 0.114, respectively). At T1, patients reported a mean treatment satisfaction, scored through a scale ranging from 0 to 10, of 8.0 ± 2.3. There was no difference in treatment satisfaction between IBSWF and IBSF groups or in different IBS subtypes (*p* = 0.407 and *p* = 0.998, respectively).

#### 3.2.3. Frequency of Symptoms Associated with Bowel Movements

We evaluated the reliability of the “bowel habits questionnaire” through Cronbach’s alpha. We obtained values > 0.7, both considering all patients together and individual subtypes of IBS. These values of alpha indicate good reliability of the questionnaire (Table 3). At T0 and at T1, there were no differences in frequency of defecation associated symptoms (evaluated with the “bowel habits questionnaire”) between IBSF and IBSWF patients (Table 3). Considering all IBS patients together, the “bowel habits questionnaire” showed a reduction at T1, compared to T0, in the following mean frequency of symptoms: abdominal pain (2.6/4 at T0 vs. 1.5/4 T1, *p* < 0.001), abdominal bloating (2.9/4 at T0 vs. 2.3/4 at T1, *p* = 0.025), and watery stools (1.9/4 at T0 vs. 1.5/4 at T1, *p* = 0.049). IBSF patients showed a significant improvement in watery stools compared to IBSWF patients. Patients labelled as IBS-D at baseline showed a significant increase in the frequency of hard stools and a decrease in the frequency of liquid stools. IBS-D patients also significantly improved in abdominal bloating (Table 3).

### 3.3. Psychological Outcomes of Long-Term AdLFD

Depressive and anxious pathological states were identified through the HADS score (Table 4). IBS-C showed higher levels of HAD-A score compared to IBS-D and IBS-M at T0 (*p* = 0.012). There were no differences in the mean HAD-A and HAD-D score between IBS subtypes at T0 and T1. Overall, there was a mean improvement of 2.3 points on the HAD-A subscale at T1. In the same way, we obtained a mean reduction of 1.1 points on the HAD-D subscale at T1 (Table 4). IBS-C and IBS-M patients reported a significant improvement at T1 in the HAD-A subscale, while IBS-D patients, despite showing a decrease in the mean score for anxiety, did not reach a level of significance.

Considering the effect of comorbid fibromyalgia, at T0, IBSF patients showed higher scores on both HAD-A and HAD-D subscales (*p* = 0.026 and *p* = 0.05, respectively). At T1, there were no differences in mean HAD-A or HAD-D scores between IBSF and IBSWF groups. There was a mean decrease of 3.7 points in HAD-A scores and 2.1 points in HAD-D scores in IBSF patients (*p* = 0.002 and *p* < 0.001, respectively) compared to T0, while IBSWF patients, even though showing a decrease in mean HAD-A and HAD-D scores, did not reach a level of significance. However, comparing the improvement rates between IBSF and IBSWF for both HAD-A and HAD-D subscales, even though IBSF groups tend to have a greater improvement compared to the IBSWF group in HAD-A score, there were no statistically significant differences (Table 4).

### 3.4. Long-Term Adherence to AdLFD

Mean FARS scores for all patients at T1 was 20.5 ± 5.2. In detail, 44 patients (86.3%) showed good long-term adherence to the AdLFD (FARS score ≥ 20 [35]). There was no difference in the FARS mean scores between IBSF and IBSWF patients (20.4 ± 5.4 vs. 20.5 ± 5.2, respectively, *p* = 0.943) or according to IBS subtype (21.1 ± 5.0 for IBS-D, 19.9 ± 5.7 for IBS-M, and 20.3 ± 5.0 for IBS-M, *p* = 0.737). It was also evaluated whether the adherence could be influenced by the follow-up time for both IBSF and IBSWF groups. There was no correlation between the two variables in the two groups (r = 0.156 and r = 0.098 for IBSF and IBSWF patients, respectively, *p* > 0.05), meaning that the length of follow-up was not related to long-term adherence to LFD in either group of patients.

### 3.5. Clinical Predictor of Long-Term Response and Adherence to AdLFD

We evaluated the presence of possible clinical factors associated with long-term response to AdLFD (Table 5). With univariate analysis, gender, age, and BMI did not influence long-term treatment response. Patients with a severe IBS showed a higher response rate compared to moderate and mild IBS patients. Regarding frequency of symptoms associated with bowel movements, respondents showed a higher frequency of watery stools, higher frequency of abdominal pain, and higher frequency of abdominal bloating at baseline. Comorbid fibromyalgia or a depressive and anxious pathological state did not influence long-term treatment response to AdLFD.

On multivariate analysis, the frequency of watery stools was independently associated with long-term response to AdLFD. Patients who more frequently had watery stools have a higher probability of responding in the long term to AdLFD. A trend, although not statistically significant, was also found with multivariate analysis regarding total IBS-SSS score and bowel dissatisfaction at T0 item of the IBS-SSS score (Table 5).

We also evaluated clinical factors associated with long-term adherence to AdLFD (assessed by means of FARS questionnaire) (Table 6). The only clinical feature that showed an association with long-term adherence was the severity of abdominal pain item on the IBS-SSS score. A comorbid fibromyalgia or depressive and anxious pathological state did not influence long-term adherence to AdLFD.

## 4. Discussion

IBS treatment is a complex challenge both for general practitioners and gastroenterologists [40]. Dietary managements are among the most prescribed therapies for IBS [41] and an LFD is currently considered a “second line” dietary management for IBS [3,4]. However, some concerns have been raised regarding LFD (e.g., intestinal dysbiosis, nutritional inadequacy, constipation, eating disorders, etc.) [33,35,42,43,44,45,46]. In order to overcome these possible limitations, a less restrictive diet based on the exclusion of only the food “triggers” that patients recognize can be designed. However, adherence to an LFD may be influenced by several factors [24]: this diet can be difficult to teach, to learn, and also to continue in the long term. Furthermore, comorbid conditions (such as fibromyalgia) may be further factors influencing long-term outcomes of this dietary strategy. In this regard, we aimed to understand if comorbid fibromyalgia influences long-term adherence to an AdLFD and identify its outcomes on gastrointestinal symptoms in patients with IBS. We evaluated the outcomes and adherence of AdLFD on 51 IBS patients, both with and without comorbid fibromyalgia (IBSF and IBSWF, respectively) (Table 1), after a mean follow-up of 62.5 ± 22.7 months.

Considering the outcomes of AdLFD on IBS symptoms, 80% of patients reported a long-term symptom relief and a mean treatment satisfaction of 8/10, irrespective of the subtype of IBS or presence of comorbid fibromyalgia. We considered long-term responders to AdLFD patients reporting, at T1, an improvement of at least 50 points on the total IBS-SSS score [31]. Particularly, 30 patients (58.8%) showed a long-term response on IBS abdominal symptoms after a mean follow-up of 62.5 ± 22.7 months. Several randomized clinical trials have provided evidence to support the use of an LFD as an effective therapeutic tool in the management of IBS [47,48,49,50,51,52,53]. Furthermore, these data on the efficacy of AdLFD are in line with a recently published study, which reported symptom relief in 60% of the patients at a mean follow-up time of 44 ± 30 months [54]. IBSF patients showed higher levels of total IBS-SSS compared to IBSWF patients at baseline (although not significant). At T1, there were no differences in mean IBS symptom severity (evaluated by IBS-SSS) between IBSF and IBSWF patients, confirming that this comorbidity did not influence AdLFD clinical outcomes (Table 2). We observed only a slight difference in IBS-SSS items among patients with and without comorbid fibromyalgia, confirming that this comorbidity did not influence in a decisive way the long-term outcomes of AdLFD on IBS symptoms.

Furthermore, we reported a long-term effect of AdLFD on stool consistency, with a significant reduction in frequency of watery stools (BSFC 6–7) in the IBS-D group of patients (Table 3). This was also highlighted by a recent study by Halmos et al. [52], which showed an improvement in stool consistency among patients with diarrhea treated with an LFD compared to a normal diet. Notably, while at T0 there were no differences in frequency of watery stools or IBS subtypes between IBSF and IBSWF, patients with fibromyalgia improved after AdLFD, showing a significant reduction in the frequency of watery stools, while IBSWF reported no change. The greater effectiveness of the LFD in patients with diarrhea (and especially with comorbid fibromyalgia, independently of the subtype of IBS) could be attributed to the osmotic nature of FODMAPs present in the normal diet, which contributes to increased fluid loss [55]. FODMAPs are poorly absorbed in the small intestine and transit to the colon, where they exert an osmotic effect, causing fluid shift in the intestinal lumen and stimulating gas production (mainly hydrogen and methane) through fermentation by colonic microbiota [55]. These processes can cause abdominal pain, bloating, flatulence, and diarrhea [24]. The greater effectiveness of the LFD in patients with diarrhea needs to be verified on a larger sample.

Regarding other symptoms associated with defecation, although not statistically significant, both IBSF and IBSWF patients reported an improvement at T1 in the frequency of abdominal bloating. We did not identify any other difference in symptoms associated with defecation at T0 or at T1 between IBSF and IBSWF patients (Table 3). We observed an increased frequency of fecal consistency in the IBS-D group and not in the IBS-M and IBS-C group. Constipation is one of the issues raised regarding an LFD and may be explained by a reduction in fiber intake if wholegrain wheat products or high FODMAP fruit and vegetables are not replaced with suitable low-FODMAP alternatives [24]. On a pathophysiological level, a reduction in FODMAPs could reduce osmotic fluid transit into the gut lumen, increasing the likelihood of constipation [24]. These effects have been thought to be particularly harmful in patients with constipation. However, our data regarding the stool consistency are encouraging and need to be confirmed by adequately powered randomized clinical trials.

Anxiety and depression levels in patients before and after the AdLFD and possible influences of comorbid fibromyalgia were also assessed (Table 4). IBSF patients showed a higher HADS score (both for anxiety and depression) at baseline compared to IBSWF patients, but no difference was observed in mean HADS score at T1. This suggests that fibromyalgia has no influence on the outcomes of the AdLFD regarding these psychological comorbidities.

Regarding long-term adherence to AdLFD, the FARS questionnaire showed that 86.3% of patients had a long-term adherence to the diet, regardless of the subtype of IBS or presence of comorbid fibromyalgia (Table 6). Our data regarding adherence to an AdLFD confirm previously published studies [33,56,57] but for a longer period of time. However, among different studies, there is no uniformity in how to assess long-term adherence, and this can make the results difficult to compare. It is likely that the high degree of satisfaction influenced the high adherence to the diet in the long term. Long-term adherence is critical to its success [58,59] and represents a predictor of clinical response [60]. Therefore, the AdLFD may overcome some of these difficulties. This implies that an AdLFD should be suggested under the supervision of an experienced nutritional healthcare professional. It ensures a diet that the patient can adhere to in the long term without jeopardizing nutritional adequacy [61].

Finally, we searched for clinical predictors of long-term response and adherence to the AdLFD (Table 5 and Table 6, respectively). We acknowledge that this is not the first study aiming to find clinical predictors of response to this diet. However, while Colmier et al. [62] in their original paper aimed to find short-term predictors of symptom-specific treatment response of LFD and Wilder-Smith et al. [63] focused on finding short-term predictors of LFD response in patients with lactose or fructose intolerance, our aim was to look for long-term predictors of symptom improvement of AdLFD. Severe IBS symptoms, higher frequency of watery stools, abdominal pain, and abdominal bloating showed as predictors of long-term response. Notably, comorbid fibromyalgia did not influence long-term response to the AdLFD. Regarding predictors of long-term adherence, the only identified clinical feature associated with long-term adherence to AdLFD was the severity of abdominal pain (Table 6). Patients showing a more severe abdominal pain at baseline were more likely to be adherent in the long term.

### Limitations of the Study

The present study has several limitations. Firstly, at T1, we conducted a telephone interview and we were able to reassess about 80% of patients. This response rate is comparable with the best results reported in the literature regarding telephone surveys [64,65]. Indeed, studies using telephone interviews or mailed questionnaires have yielded a response rate ranging from 19% to 80% [64,65]. Even paradoxically considering all the dropouts as non-responders, the percentage of patients who reported a beneficial effect of the AdLFD would be about 60%. This percentage would, however, be somewhat better than the results obtained in many trials, as well as those with new and expensive drugs for IBS [66]. Secondly, it was not possible to take into account the effects of the severity of fibromyalgia on the results. This was mainly due to the nature of the T1 evaluation (phone interview), which did not allow us to assess fibromyalgia symptoms properly or to perform a physical examination of the patients. However, this was beyond the scope of our study and, moreover, the enrolled patients were referred to an LFD for management of IBS gastrointestinal symptoms and not for fibromyalgia symptoms. In addition, as our study was monocentric, we were able to enroll a relatively low number of patients in our study. Long-term studies with a higher number of patients are needed to confirm our results. Furthermore, the possible effects of drugs assumed in this period of time were not evaluated but, being quite a long period, this was effectively practically impossible to achieve. However, beyond these limitations, we believe that the results obtained can offer interesting and useful insights to guide future research.

## 5. Conclusions

The results of the present study confirmed the long-term positive effect of an AdLFD on the improvement of IBS symptoms, with some positive effects also on anxiety–depressive symptomatology. Comorbid fibromyalgia had an only slight influence on the long-term outcomes of the AdLFD on IBS symptoms, without impacting on global symptom relief and with no influence on long-term adherence to the AdLFD. Severe IBS symptoms, higher frequency of watery stools, abdominal pain, and abdominal bloating were predictors of long-term response of the AdLFD. Having comorbid fibromyalgia was not a predictive factor of better or worse long-term response nor adherence to the AdLFD. Therefore, this dietetic approach can be taken into account also in fibromyalgia patients for a nonpharmacological management of IBS symptoms, even if multicentric studies on a larger sample would be welcome to confirm our findings.

## Figures and Tables

**Table 1:** Demographic characteristics of patients at T0. Statistics: frequency (%) or mean (±SD).

Variables	IBSWF Group (*n* = 32)	IBSF Group (*n* = 19)	*p*-Value
Gender			0.928
M (*n* = 7)	4 (12.5%)	3 (15.8%)	
F (*n* = 44)	28 (87.5%)	16 (84.2%)	
Age	51.7 ± 15.6	55.3 ± 8.2	0.361
BMI	23.7 ± 4.0	25.9 ± 4.2	0.071
IBS subtypes			0.281
IBS-D	13 (40.6%)	10 (52.6%)	
IBS-C	12 (37.5%)	8 (42.1%)	
IBS-M	7 (21.9%)	1 (5.26%)	

Abbreviations: IBSWF = IBS patients without comorbid fibromyalgia; IBSF = IBS patients with comorbid fibromyalgia; IBS = irritable bowel syndrome; BMI = body mass index.

**Table 2:** IBS-SSS total score and single items between T0 and T1 in all IBS patients and in subgroups according to the presence or absence of fibromyalgia. Statistics: mean (±SD).

	Groups	T0	T1	*p*-Value ^1^	*p*-Value Inter-Assay ^2^
IBS-SSS total score	All patients (*n* = 51)	313.5 ± 95	220.1 ± 112	<0.001	
	IBSWF patients (*n* = 32)	309.9 ± 80.5	225.2 ± 128	0.002	0.588
	IBSF patients (*n* = 19)	319.6 ± 118.9	211.58 ± 80.6	0.007	
Severity of abdominalpain	All patients (*n* = 51)	56.2 ± 26.1	40.2 ± 33.3	0.008	
	IBSWF patients (*n* = 32)	59.1 ± 23.2	41.7 ± 34.1	0.005	0.753
	IBSF patients (*n* = 19)	51.3 ± 30.4	37.63 ± 32.8	0.25	
Days with abdominalpain	All patients (*n* = 51)	54.9 ± 30.1	27.8 ± 26.4	<0.001	
	IBSWF patients (*n* = 32)	52.2 ± 28.7	29.4 ± 28.7	0.005	0.34
	IBSF patients (*n* = 19)	59.5 ± 32.7	25.3 ± 22.5	0.001	
Severity of abdominaldistention	All patients (*n* = 51)	61.2 ± 23.7	50.4 ± 34.7	0.069	
	IBSWF patients (*n* = 32)	63.4 ± 21.8	48.8 ± 37.3	0.024	0.365
	IBSF patients (*n* = 19)	57.5 ± 26.8	53.2 ± 30.6	0.675	
Bowel habit dissatisfaction	All patients (*n* = 51)	71.4 ± 29.9	48.5 ± 33.2	<0.001	
	IBSWF patients (*n* = 32)	66.9 ± 31.0	46.1 ± 36.2	0.029	0.684
	IBSF patients (*n* = 19)	78.8 ± 27.0	52.6 ± 27.9	0.002	
IBS interference with lifestyle item	All patients (*n* = 51)	70.2 ± 26.1	53.1 ± 34.1	0.006	
	IBSWF patients (*n* = 32)	68.4 ± 24.6	59.2 ± 33.7	0.165	0.081
	IBSF patients (*n* = 19)	73.1 ± 28.8	42.9 ± 33.2	0.007	

^1^*t*-test for repeated measures; ^2^ ANOVA for repeated measures. Abbreviations: IBSWF = IBS patients without comorbid fibromyalgia; IBSF = IBS patients with comorbid fibromyalgia; IBS = irritable bowel syndrome; IBS-SSS = IBS Symptom Severity Score.

**Table 3:** “Bowel habits questionnaire” outcomes before and after the AdLFD according to IBS subtype and presence of fibromyalgia. Statistics: mean (±SD).

Bowel Habits Questionnaire Items		IBS-Subtype				Comorbid Fibromyalgia		
		IBS-D	IBS-C	IBS-M	*p*-Value ^1^	Yes	No	*p*-Value ^1^
Straining at defecation	T0	0.8 ± 1.0	3.4 ± 0.9	1.6 ± 1.4	<0.001 ^#^	1.4 ± 1.4	1.6 ± 1.5	0.742
	T1	0.8 ± 1.2	3.1 ± 1.0	1.5 ± 1.2	<0.001 ^#^	1.4 ± 1.3	1.5 ±1.5	0.806
	*p*-value ^2^	0.788	0.563	0.732		0.889	0.669	
Incomplete evacuation	T0	1.4 ± 1.2	2.9 ± 1.5	1.7 ± 1.4	0.069	1.7 ± 1.3	1.8 ± 1.5	0.811
	T1	1.3 ± 1.5	1.6 ± 1.8	1.7 ± 1.5	0.749	1.2 ± 1.4	1.4 ± 1.6	0.464
	*p*-value ^2^	0.732	0.129	0.886		0.65	0.2	
Painful defecation	T0	0.8 ± 1.3	2.9 ± 1.1	0.7 ± 1.4	<0.001 ^#^	1.2 ± 1.4	1.0 ± 1.4	0.612
	T1	0.8 ± 1.2	2.0 ± 1.4	0.7 ± 1.0	0.089	1.1 ± 1.2	0.9 ± 1.2	0.519
	*p*-value ^2^	1.0	0.247	0.815		0.650	0.587	
Hard stools (BSFC 1–2)	T0	0.4 ± 0.5	3.0 ± 0.8	1.1 ± 1.3	<0.001 ^+^	1.1 ± 1.0	1.1 ± 1.4	0.908
	T1	1.3 ± 1.4	2.8 ± 1.4	1.1 ± 1.1	0.022 ^=^	1.3 ± 1.2	1.6 ± 1.5	0.463
	*p*-value ^2^	0.006	0.598	0.867		0.674	0.054	
Watery stools (BSFC 6–7)	T0	3.0 ± 0.9	0.6 ± 0.5	1.3 ± 0.9	<0.001 ^@^	2.3 ± 1.4	1.8 ± 1.2	0.171
	T1	2.0 ± 1.3	0.5 ± 0.8	1.4 ± 1.3	0.002 ^$^	1.2 ± 1.3	1.7 ± 1.3	0.383
	*p*-value ^2^	0.007	0.685	0.748		0.007	0.716	
Fragmented defecation	T0	1.8 ± 0.9	2.1 ± 1.3	1.4 ± 1.3	0.37	1.5 ± 1.2	1.8 ± 1.4	0.375
	T1	1.3 ± 1.3	2.6 ± 1.7	1.6 ± 1.2	0.19	1.6 ± 1.2	1.7 ± 1.5	0.849
	*p*-value ^2^	0.077	0.606	0.56		0.65	0.648	
Defecatory urgency	T0	2.2 ± 1.3	1.5 ± 1.6	1.3 ± 1.0	0.069	1.7 ± 1.5	1.6 ± 1.2	0.973
	T1	2.0 ± 1.3	1.0 ± 1.4	1.6 ± 1.2	0.229	1.6 ± 1.3	1.6 ± 1.3	0.655
	*p*-value ^2^	0.496	0.227	0.356		0.667	1.00	
Incontinence for gas and/or feces	T0	1.3 ± 1.3	1.4 ± 0.9	1.0 ± 0.9	0.534	1.0 ± 1.0	1.3 ± 1.1	0.215
	T1	1.0 ± 1.4	1.5 ± 1.9	1.4 ± 1.6	0.563	1.2 ± 1.6	1.2 ± 1.5	0.722
	*p*-value ^2^	0.383	0.836	0.226		0.32	0.544	
Abdominal pain	T0	2.8 ± 1.0	3.0 ± 0.0	2.2 ± 1.1	0.057	2.6 ± 1.2	2.6 ± 0.9	0.876
	T1	1.5 ± 1.4	2.0 ± 1.2	1.4 ± 1.4	0.503	1.2 ± 0.9	1.7 ± 1.3	0.147
	*p*-value ^2^	<0.001	0.05	0.057		<0.001	0.003	
Abdominal bloating	T0	3.0 ± 1.0	3.1 ± 1.1	2.7 ± 1.1	0.604	3.0 ± 0.9	2.8 ± 1.1	0.536
	T1	2.1 ± 1.4	2.8 ± 1.6	2.2 ± 1.4	0.625	2.3 ± 1.4	2.2 ± 1.4	0.815
	*p*-value ^2^	0.039	0.654	0.234		0.055	0.116	

^1^ Between groups at T0 and at T1; ^2^ between T0 and T1 in IBS subtypes and patients with and without fibromyalgia; ^#^ IBS-D vs. IBS-C *p* < 0.01 and IBS-D vs. IBS-M *p* < 0.01; ^+^ IBS-D vs. IBS-C *p* < 0.01, IBS-M vs. IBS-C *p* < 0.01, and IBS-D vs. IBS-M *p* < 0.05; ^@^ IBS-D vs. IBS-C *p* < 0.01 and IBS-D vs. IBS-M *p* < 0.01; ^=^ IBS-C vs. IBS-D *p* < 0.05 and IBS-C vs. IBS-M *p* < 0.01; ^$^ IBS-C vs. IBS-D *p* = 0.01. Abbreviations: IBS = irritable bowel syndrome; IBS-D = IBS with predominant diarrhea; IBS-C = IBS with predominant constipation IBS-M = IBS with mixed bowel habits; BSFC= Bristol stool form chart. Cronbach’s alpha for the questionnaire in all patients is 0.736. Cronbach’s alpha for IBS-D patients’ subgroup is 0.708, for IBS-C patients’ subgroup is 0.755, and for IBS-M patients’ subgroup is 0.785.

**Table 4:** Comparisons of HAD-A and HAD-D scores between T0 and T1 in all patients and divided according to IBS subtype and comorbid fibromyalgia. Statistics: mean (±SD).

	HAD-A Scores T0 (*n* = 51)	HAD-A Scores T1 (*n* = 51)	*p*-Value ^1^	*p*-Value Inter-Assay ^2^
All patients (*n* = 51)	9.4 ± 4.1	7.1 ± 4.1	<0.001	
IBS Subtype				
IBS-D (*n* = 23)	7.9 ± 4.3	6.3 ± 3.6	0.129	
IBS-C (*n* = 8)	12.9 ± 3.2	8.1 ± 3.5	0.005	
IBS-M (*n* = 20)	9.7 ± 3.2	7.6 ± 4.8	0.024	
IBSF patients (*n* = 19)	11.0 ± 3.3	7.3 ± 4.9	0.002	0.078
IBSWF patients (*n* = 32)	8.4 ± 4.2	6.9 ± 3.6	0.19	
	HAD-D scores T0 (*n* = 51)	HAD-D scores T1 (*n* = 51)		
All patients (*n* = 51)	6.9 ± 3.9	5.8 ± 4.3	0.04	
IBS Subtype				
IBS-D (*n* = 23)	5.9 ± 4.4	4.5 ± 4.1	0.129	
IBS-C (*n* = 8)	8.9 ± 3.9	7.9 ± 5.4	0.465	
IBS-M (*n* = 20)	7.3 ± 3.0	6.5 ± 5.4	0.279	
IBSF patients (*n* = 19)	8.3 ± 3.6	6.2 ± 4.1	<0.001	0.135
IBSWF patients (*n* = 32)	6.1 ± 3.8	5.6 ± 4.5	0.398	

^1^*t*-test for repeated measures; ^2^ ANOVA for repeated measures. Abbreviations: IBS = irritable bowel syndrome; IBS-D = IBS with predominant diarrhea; IBS-C = IBS with predominant constipation; IBS-M = IBS with mixed bowel habits; IBSWF = IBS patients without comorbid fibromyalgia; IBSF = IBS patients with comorbid fibromyalgia; HAD-A = Hospital Anxiety and Depression—Anxiety score; HAD-D = Hospital Anxiety and Depression—Depression score.

**Table 5:** Patients’ features at T0 and their association with long-term response to AdLFD, assessed by univariate analysis and multivariate analysis using the stepwise method. Statistics: frequency (%) or mean (±SD).

Patients’ Features	Univariate			Multivariate	
	Responders (*n* = 30)	Non-Responders (*n* = 21)	*p*-Value ^1^	OR (95% CI)	*p*-Value ^2^
Gender			0.355		
Male (*n* = 7)	3 (10%)	4 (19.1%)			
Female (*n* = 44)	27 (90%)	17 (80.9%)			
Age	52.5 ± 12.2	53.8 ± 14.9	0.738		
BMI	24.5 ± 4.8	24.6 ± 3.4	0.958		
IBS Subtype			0.396		
IBS-D (*n* = 23)	16 (53.3%)	7 (33.3%)			
IBS-C (*n* = 8)	5 (16.7%)	3 (14.3.8%)			
IBS-M (*n* = 20)	9 (30.0%)	11 (52.4%)			
IBS-SSS at T0					
T0 IBS-SSS total score	355.1 ± 80.2	254.2 ± 84.9	<0.001		0.079
T0 Severity of abdominal pain item	61.8 ± 21.5	48.1 ± 30.2	0.064		
T0 Days with abdominal pain item	67.0 ± 28.4	37.6 ± 23.9	<0.001		0.167
T0 Severity of abdominal distention item	65.9 ± 22.9	54.4 ± 23.6	0.088		
T0 Bowel habit dissatisfaction item	82.9 ± 21.8	54.9 ± 32.5	<0.001		0.054
T0 IBS interference with lifestyle item	77.5 ± 24.6	59.7 ± 25.0	0.015		0.568
IBS-SSS severity categories at T0			<0.001		0.626
Mild + Moderate (75–300)	7 (23.3%)	16 (76.2%)			
Severe (301–500)	23 (76.7%)	5 (23.8%)			
Comorbid fibromyalgia			0.917		
Yes (*n* = 19)	11 (36.7%)	8 (38.1%)			
No (*n* = 32)	19 (63.3%)	13 (61.9%)			
“Bowel habits questionnaire”					
T0 Straining at defecation	1.7 ± 1.6	1.3 ± 1.3	0.365		
T0 Incomplete evacuation	2.0 ± 1.5	1.4 ± 1.1	0.117		
T0 Painful defecation	1.3 ± 1.5	0.7 ± 1.1	0.124		
T0 Hard stools (BSFC 1–2)	1.1 ± 1.3	1.0 ± 1.2	0.714		
T0 Watery stools (BSFC 6–7)	2.4 ± 1.3	1.3 ± 1.0	0.002	2.7 (1.3–5.7)	0.010
T0 Fragmented defecation	1.7 ± 1.3	1.6 ± 1.3	0.761		
T0 Defecatory urgency	1.9 ± 1.4	1.5 ± 1.3	0.323		
T0 Incontinence for gas and/or feces	1.2 ± 1.2	1.2 ± 0.9	0.821		
T0 Abdominal pain	3.0 ± 0.9	2.1 ± 1.0	0.002		0.796
T0 Abdominal bloating	3.2 ± 0.8	2.4 ± 1.1	0.003		0.726
HADS score					
T0 HAD-A	9.2 ± 4.0	9.6 ± 4.2	0.773		
T0 HAD-D	6.8 ± 4.1	7.1 ± 3.7	0.769		

^1^ Univariate *p*-Value; ^2^ Multivariate *p*-Value. Abbreviations: IBS = irritable bowel syndrome; IBS-D = IBS with predominant diarrhea; IBS-C = IBS with predominant constipation; IBS-M = IBS with mixed bowel habits; BMI = body mass index; BSFC = Bristol stool form chart; IBS-SSS = IBS symptom severity score; HADS = Hospital Anxiety and Depression Scale; HAD-A = Hospital Anxiety and Depression—Anxiety score; HAD-D = Hospital Anxiety and Depression—Depression score.

**Table 6:** Patients’ features at T0 and their association with long-term adherence to AdLFD. Statistics: frequency (%) or mean (±SD).

Patients’ Features	Adherent (*n* = 44)	Non-Adherent (*n* = 7)	*p*-Value ^1^
Gender			0.573
Male (*n* = 7)	7 (15.9%)	0 (0.0%)	
Female (*n* = 44)	37 (84.1%)	7 (100.0%)	
Age	53.1 ± 13.4	52.6 ± 13.4	0.918
BMI	24.6 ± 4.3	24.3 ± 3.9	0.872
IBS Subtype			0.257
IBS-D (*n* = 23)	20 (45.5%)	3 (42.9%)	
IBS-C (*n* = 8)	7 (15.9%)	1 (14.3%)	
IBS-M (*n* = 20)	17 (38.6%)	3 (42.9%)	
IBS-SSS at T0			
T0 IBS-SSS total score	304.9 ± 91.6	367.9 ± 110.8	0.106
T0 Severity of abdominal pain item	52.8 ± 24.4	77.1 ± 28.7	0.02
T0 Days with abdominal pain item	54.1 ± 30.6	60.0 ± 28.9	0.635
T0 Severity of abdominal distention item	58.9 ± 22.9	75.7 ± 25.1	0.081
T0 Bowel habit dissatisfaction item	70.2 ± 28.5	78.6 ± 39.3	0.497
T0 IBS interference with lifestyle item	69.2 ± 24.0	76.4 ± 38.4	0.499
IBS-SSS severity categories at T0			0.573
Mild (75–175)	3 (6.8%)	0 (0.0%)	
Moderate (176–300)	18 (40.9%)	2 (28.6%)	
Severe (301–500)	23 (52.3%)	5 (71.4%)	
Comorbid fibromyalgia			0.928
Yes (*n* = 19)	16 (36.4%)	3 (42.9%)	
No (*n* = 32)	28 (63.6%)	4 (57.1%)	
“Bowel habits questionnaire”			
T0 Straining at defecation	1.5 ± 1.4	1.6 ± 1.8	0.906
T0 Incomplete evacuation	1.7 ± 1.4	1.9 ± 1.7	0.82
T0 Painful defecation	1.0 ± 1.3	1.6 ± 2.0	0.325
T0 Hard stools (BSFC 1–2)	1.1 ± 1.2	1.1 ± 1.7	0.886
T0 Watery stools (BSFC 6–7)	1.9 ± 1.2	2.0 ± 1.6	0.898
T0 Fragmented defecation	1.6 ± 1.2	2.0 ± 1.9	0.499
T0 Defecatory urgency	1.8 ± 1.3	1.4 ± 1.5	0.502
T0 Incontinence for gas and/or feces	1.2 ± 1.1	1.0 ± 1.4	0.615
T0 Abdominal pain	2.6 ± 1.0	3.0 ± 1.2	0.269
T0 Abdominal bloating	2.9 ± 1.0	3.0 ± 1.3	0.749
HADS score			
T0 HAD-A	9.1 ± 3.6	10.9 ± 6.3	0.302
T0 HAD-D	6.9 ± 3.9	7.0 ± 3.7	0.943

^1^ Univariate *p*-value. Abbreviations: IBS = irritable bowel syndrome; IBS-D = IBS with predominant diarrhea; IBS-C = IBS with predominant constipation; IBS-M = IBS with mixed bowel habits; BMI = body mass index; IBS-SSS = IBS symptom severity score; BSFC = Bristol stool form chart; IBS-SSS = IBS symptom severity score; HADS = Hospital Anxiety and Depression Scale; HAD-A = Hospital Anxiety and Depression—Anxiety score; HAD-D = Hospital Anxiety and Depression—Depression score.

## Data Availability

The data presented in this study are available on request from the corresponding author (the data are not publicly available due ethical restrictions).

## Supplementary Materials

The following supporting information can be downloaded at: https://www.mdpi.com/article/10.3390/nu16193419/s1, Table S1: Low-FODMAP diet advice used in the restriction phase of the diet (first 8 weeks); Table S2: Categories of FODMAPs mainly excluded by patients in the AdLFD. The more frequently excluded categories were lactose, fructose, and fructans, with no differences in the frequency of exclusion between IBSWF and IBSF groups.
