# Supplementary material for: Adapted Low-FODMAP Diet in IBS Patients with and without Fibromyalgia: Long-Term Adherence and Outcomes
Source: Nutrients. 2024 Oct 9;16(19):3419. doi: 10.3390/nu16193419 (PMC11478509; doi:10.3390/nu16193419)
Supplement: Supplementary file 1 [file nutrients-16-03419-s001.zip › nutrients-3220232-supplementary.pdf]

Table S1. Low FODMAP diet advices used in the restriction phase of the diet (first 8 weeks).

Food	Allowed foods	Forbidden foods
**Cereals**	Rice, porridge, oats, quinoa, tapioca, millet, amaranth, buckwheat, bread and gluten-free cereal, potato flour	Bread and bakery products, biscuits, croissants, pasta, wheat flour, Kamut, barley, rye, couscous, flour, muesli
**Milk and derivatives**	Lactose-free milk, rice milk, oat milk, soy milk and all vegetable drinks, yogurt lactose-free, soy yogurt, Greek yogurt, fruit sorbets, hard cheeses	Cow milk, goat milk, yogurt with lactose, fresh cheeses, ice cream, cream
**Dried fruits**	Almonds, hazelnuts, walnuts, pine nuts	Pistachios, cashews
**Vegetables**	Carrots, pumpkin, Chinese cabbage, celery, lettuce, spinach, potatoes, tomatoes, zucchini, eggplant, green beans, red pepper, herbs, olives, bamboo shoots, fresh herbs	Asparagus, cauliflower, garlic, onion, shallot, mushrooms, leek, chicory, fennel, artichokes, Brussel sprouts, broccoli, radishes, peppers, turnips, Jerusalem artichokes
**Legumes**	Peas	Beans, chickpeas, lentils, soybeans
**Fruit**	Banana, blueberry, strawberry, raspberry, grape, melon, grapefruit, kiwi, oranges, lemons, limes, pineapple, passion fruit	Apple, pear, watermelon, mango, apricot, avocado, cherries, peaches, plums, persimmon, lychee, fruit juices
**Sweeteners**	White sugar, brown sugar, maple syrup	Agave, honey, fructose, xylitol, maltitol, mannitol, sorbitol

Table S2. Categories of FODMAPs mainly excluded by patients in the AdLFD. The more frequently excluded categories were lactose, fructose and fructans, with no differences in the frequency of exclusion between IBSWF and IBSF groups. Statistics: frequency (%) or mean (±SD).

Excluded FODMAP category		IBSWF group (n=32)	IBSF group (n=19)	p-value
Lactose exclusion	No	5 (15.6%)	5 (26.3%)	0.47
	Yes	27 (84.4%)	14 (73.7%)	
Fructose exclusion	No	14 (43.8%)	7 (36.8%)	0.77
	Yes	18 (56.2%)	12 (63.2%)	
Fructans exclusion	No	12 (37.5%)	7 (36.8%)	1
	Yes	20 (62.5%)	12 (63.2%)	
Galactans exclusion	No	26 (81.3%)	16 (84.2%)	1
	Yes	6 (18.7%)	3 (15.8%)	
Polyols exclusion	No	22 (68.8%)	17 (89.5%)	0.17
	Yes	10 (31.2%)	2 (10.5%)	
